# IFCC recommendation: The theory of reference values. Part 4. Control of analytical variation in the production, transfer and application of reference values

**DOI:** 10.1155/S146392469100038X

**Published:** 1991

**Authors:** Helge Erik Solberg, D. Stamm

**Affiliations:** 1 Department Clinical Chemistry Rikshospitalet Oslo 1 Oslo N-0027 Norway; 2 Department of Clinical Chemistry Max-Planck Institute of Psychiatry Munich Germany

## Abstract

This paper is the fourth in a series of Recommendations on the Theory of Reference Values. The others cover:

Part 1. The Concept of Reference Values [1].

Part 2. Selection of Individuals for the Production of Reference Values [2].

Part 3. Preparation of Individuals and Collection of Specimens for the Production of Reference Values [3].

Part 5. Statistical Treatment of Collected Reference Values. Determination of Reference Limits [4].

Part 6. Presentation of Observed Values Related to Reference Values [5].

A Guide to the Documents is currently in preparation.

The Expert Panel of Theory of Reference Values (EPTRV) was created in 1970 by the Committee on Standards (at present: Scientific Division) of the International Federation of Clinical Chemistry (IFCC). Its task was to develop a nomenclature and recommend procedures for the production of reference values and their treatment, and presentation of observed values in relation to reference data.

The first document in the above-mentioned series describes the subject of reference values and defines various terms. It would be best to read it for a thorough understanding of the present document.


Journal of Automatic Chemistry, Vol. 13, No. 5 (September–October 1991), pp. 231–234

*International Federation of Clinical Chemistry (IFCC)*

### IFCC recommendation: The theory of reference values. Part 4. Control of analytical variation in the production, transfer and application of reference values*

**Helge Erik Solberg**
*Department Clinical Chemistry, Rikshospitalet, N-0027 Oslo 1, Oslo, Norway*

**and D. Stamm**
*Department of Clinical Chemistry, Max-Planck Institute of Psychiarty, Munich, Germany*

*This paper is the fourth in a series of Recommendations on the Theory of Reference Values. The others cover:*

*Part 1. The Concept of Reference Values [1].*
*Part 2. Selection of Individuals for the Production of Reference Values [2].*
*Part 3. Preparation of Individuals and Collection of Specimens for the Production of Reference Values [3].*
*Part 5. Statistical Treatment of Collected Reference Values. Determination of Reference Limits [4].*
*Part 6. Presentation of Observed Values Related to Reference Values [5].*

*A Guide to the Documents is currently in preparation.*

*The Expert Panel of Theory of Reference Values (EPTRV) was created in 1970 by the Committee on Standards (at present: Scientific Division) of the International Federation of Clinical Chemistry (IFCC). Its task was to develop a nomenclature and recommend procedures for the production of reference values and their treatment, and presentation of observed values in relation to reference data.*

*The first document in the above-mentioned series describes the subject of reference values and defines various terms. It would be best to read it for a thorough understanding of the present document.*

### 1. Introduction

### 1.1. *Aim of the document*

Observed values may be compared with reference values or with the reference distribution, reference limits and reference interval derived from the reference values [1,5]. The purpose of this document is to provide guidance on the requirements necessary to ensure that the reference values are produced using accurate and precise methods. Failure to employ methods that have known and suitably low levels of inaccuracy and imprecision will reduce the clinical utility of the results.

### 1.2. *Perspective*

It is important to establish and maintain a realistic awareness of the contribution that analytical inaccuracy and imprecision may have on the production of reference values. Biological variance is an important factor to take into consideration when determining allowable imprecision and inaccuracy [6–8]. Thus, serious efforts should be made to establish the tolerable levels of analytical inaccuracy and imprecision for the intended uses of the reference values. For example, the Aspen Conference defined in 1976 the tolerable levels of imprecision for measurements of calcium and urea in serum to be quite different, 0·9% and 6·2% (CV), respectively [6].

This document assumes that an adequate effort has been made to reduce biological variation when selecting subjects [2] and collecting specimens [3].

### 1.3. *Need for reliable methods*

The method, including the reagents and equipment used, must be described in enough detail that adequately-trained analysts will all proceed in the same manner and obtain reproducible results. The assessment of these methods should be made according to Part 2 of the recommendations of the IFCC's Expert Panel on Nomenclature and Principles of Quality Control [9].

### 1.4. *Need for long-term stability of the analytical method*

The development of reference values is a time-consuming and costly exercise. As a result, it is essential that the values must have application over a lengthy period. Furthermore, the true clinical utility of the values in diagnosis, comparative assessment and long-term monitoring necessitates the use of well-controlled analytical procedures.

Reprint requests and enquiries to: Dr Helge Erik Solberg.
* Members of the Expert Panel on the Theory of Reference Values during the preparation of this recommendation were as follows: R. Gräsbeck (FI) 1970–1978 (Chairman 1975–1978), N. Montalbetti (IT) 1982–1986, C. PetitClerc (CA) 1977–1986, H. E. Solberg (NO) 1977–1986 (Chairman since 1979), G. Siest (FR) 1973–1979, D. Stamm (DE) 1979–1986, P. Wilding (US) 1976–1984.

© IFCC 1991

### 1.5. *Experimental design*

In the production of reference values it is desirable to keep analytical variance of approximately the same magnitude as that obtained by good-quality routine analysis. Therefore, it is strongly recommended that the measurements for the production of reference values be made under strictly controlled, but realistic routine conditions, including the day-to-day imprecision.

### 1.6. *Transferability*

The complexity, cost and effort of establishing reference values are often minimal when compared with the problem of obtaining a sufficient number of adequate specimens for the production of the reference values. As a result it is often necessary to transfer reference values from one institution to another. In this case it is essential that the laboratories involved obtain comparable results; this can be achieved by assessment of the accuracy and precision of the analytical method in use through long-term inter-laboratory studies [10].

Comparability of results is also necessary for laboratories receiving specimens from mobile populations. It may be best achieved using similar analytical procedures. Should they be different, it is important that they conform to tolerable limits of inaccuracy and imprecision.

### 2. Monitoring procedures

2.1. The theoretical principles underlying quality control (QC) and the various forms that QC can take are treated in detail in the recommendation of the Expert Panel on Nomenclature and Principles of Quality Control in Clinical Chemsitry [9].

There are numerous QC procedures which will monitor the accuracy and precision of the analyses, and ensure the transferability of the data. This section provides elementary guidance in QC procedures for this purpose. It is assumed that laboratories performing this work will have certain materials and facilities available which will, probably, be in use routinely. However, it is essential that the following conditions are met:

The *calibration material* must be carefully defined [9,11], keeping in mind the presence of nonspecfic components which may contribute to the reading.

The *basis for calculation*, for example molar lineic absorbance [9,11], must be carefully defined and tested.

Suitable *control specimens* must be available commercially or be prepared by the user. They should have a matrix that has properties similar to those of the specimens used for the production of reference values [7–11]. To ensure proper monitoring of accuracy, precision and reliability of a method, selected control materials should be validated [10].

The *control procedure* should be described in detail. This description should also include decision rules for out-of-control situations and reactions to alarms [12].

### 2.2. *Recommendation for single laboratories*

In most cases, a simple system involving control specimens is recommended for this purpose [9]. The recommended system [13,14] consists of internal and external quality control procedures, see figure 1. (External quality control is also called 'external quality assessment'.)

The procedures for the two separate kinds of internal control, control of precision and control of accuracy, are shown in figure 2.

In accuracy control, it is important that the whole clinically relevant interval of measurement be monitored.

In some cases control rules are needed that are more sensitive to inaccuracy and imprecision than those described in figures 1 and 2. For example, it is important to detect small increases in inaccurcy and imprecision of the analysis of the concentration of calcium in serum because of the small biological variance of this component. Westgard, Groth and coworkers have developed control systems that help to ensure that the analytical quality is within stated limits [12,15].

### 2.3. *Recommendation for multiple laboratory collaboration*

If several laboratories are to participate in the production of reference values, the comparability of the results from these laboratories must first be evaluated. If comparability is inadequate, appropriate changes must be made in the analytical system. Whenever several laboratories are involved, there must be continuing checks on comparability while reference values are being produced.

Prior to analysing the specimens from the reference individuals, the laboratories should assess the comparability of their analytical results by using the same control material. Where stability of the component permits, it is also advisable to check comparability by exchanging human specimens. An effective model for conducting and evaluating such a long-term survey has been developed, and experience with the model has been reported [10]. If

*Internal quality control*

Control of precision
- In every run of analyses, even if the run consists of only one patient specimen
- with precision control specimen

Control of inaccuracy
- in at least every fourth run
- with accuracy control specimen

*External quality control* (or assessment)

Interlaboratory surveys
- at least three times a year for each type of quantity
- specimens shipped by the chief investigator
- evaluation on the basis on analytical results from independent reference laboratories

*Figure 1. Basic program for quality control.*

| | *Control of precision* | *Control of accuracy* |
|---|---|---|
| *Frequency* | In every run of analyses | In every fourth run |
| *Materials* | | One of a number of different accuracy |
| Control specimens | A precision control specimen from a batch used for as long a time as possible | control specimens kept on hand |
| | Concentration at the decision limit | Assigned values at normal and pathological levels |
| Monitoring device | | |
| Control chart | Form for assessment of accuracy | |
| *Task of analyst* | Recognizes control specimen<br>Knows concentration<br>Conducts duplicate determinations | Recognizes control specimen<br>Does not know concentration (known to control supervisor of the laboratory) |
| *Goals* | Assessment of random variation (random errors)<br>Detection of trends | Detection of systematic deviations (systematic errors)<br>Monitoring over the whole diagnostically relevant interval<br>Detection of influence of other components (matrix)<br>Elimination of intentional or unintentional misinterpretation of data by analyst |

*Figure 2. Recommended basic internal quality control.*

the data from the comparison study shows that no significant effect on the size of the reference interval will be the result, then a multi-laboratory study can proceed.

### 3. Transfer of reference value data

The diversity of analytical procedures has complicated the establishment of transferable reference values. Therefore, using the QC procedures above with well operated and documented methodology is a prerequisite for the transfer of reference value data from one laboratory to another. However, it is important to realize that the clinical environment is dynamic, and that reference values and associated data may become invalid or inappropriate over a period of time. To prevent the obsolescence of reference value data it is imperative to ensure the following:

(*a*) Monitor changes in method accuracy and precision.

(*b*) Check relevance of reference value data with the individuals and/or groups being assessed.

(*c*) Store the original reference value data.

(*d*) Update reference value information when necessary.

### 4. Comment

Figures 1 and 2 are intended for guidance and do not constitute the only effective methods of controlling and monitoring analytical variation.

### 5. Appendix: Methods of quality control

A simple procedure for the control of accuracy and precision is shown in figures 1 and 2.

### 5.1. *Accuracy control*

It is essential to detect changes in the calibration curve over time and to monitor the accuracy of the analytical method. This requires a number of accuracy control specimens at both normal and pathological concentrations. These control specimens shoud contain different kinds of matrix, and should also contain other components that might disturb the analysis. Accuracy and specificity will not be monitored adequately if the different concentrations of the target component are obtained by spiking the same matrix, or by adding different amounts of diluent to the same amount of the specimen during reconstitution.

### 5.2. *Precision control*

To ensure that the required precision is maintained and to detect trends, it is recommended that an adequate number of control specimens be included at fixed or random positions in each analytical run [7,8]. Ideally, precision control should be employed at different concentrations. If only one precision control is used, the concentration should match the clinical discrimination level.

### References

1. Solberg, H. E., Approved Recommendation (1986) on the theory of reference values. Part 1. The concept of reference values. *Clinica Chimica Acta,* **165** (1987), 111–118; *Journal of Clinical Chemistry and Clinical Biochemistry,* **25** (1987), 337–

342; *Annals of Biological Chemistry*, **45** (1987), 237–241; *Labmedica*, **4** (1987), 27–31.

2. PetitClerc, C. and Solberg, H. E., Approved recommendation (1987) on the theory of reference values. Part 2. Selection of individuals for the production of reference values. *Journal of Clinical Chemistry and Clinical Biochemistry*, **25** (1987), 639–644; *Clinica Chimica Acta*, **170** (1987), S3–S12.

3. Solberg, H. E. and PetitClerc, C., Approved recommendation (1988) on the theory of reference values. Part 3. Preparation of individuals and collection of specimens for the production of reference values. *Journal of Clinical Chemistry and Clinical Biochemistry*, **26** (1988), 593–598; *Clinica Chimica Acta*, **177** (1988), S1–S12.

4. Solberg, H. E., Approved recommendation (1987) on the theory of reference values. Part 5. Statistical treatment of collected reference values. Determination of reference limits. *Journal of Clinical Chemistry and Clinical Biochemistry*, **25** (1987), 645–656; *Clinica Chimica Acta*, **170** (1987), S13–S32.

5. Dybkaer, R. and Solberg, H. E., Approved recommendation (1987) on the theory of reference values. Part 6. Presentation of observed values related to reference values. *Journal of Clinical Chemistry and Clinical Biochemistry*, **25** (1987), 657–662; *Clinica Chimica Acta*, **170** (1987), S33–S42; *Labmedica*, **5** (1988), 27–30.

6. Fraser, C. G., Desirable performance standards for clinical chemistry tests. *Advances in Clinical Chemistry*, **23** (1983), 299–339.

7. Stamm, D., A new concept for quality control of clinical laboratory investigations in the light of clinical requirements and based on reference method values. *Journal of Clinical Chemistry and Clinical Biochemistry*, **20** (1982), 817–824.

8. Stamm, D., Quality control in clinical laboratories. *Proceedings of the International Congress of Clinical Chemistry, The Hague June–July 1987*, Plenum Press, New York.

9. Buttner, J., Borth, R., Boutwell, J. H., Broughton, P. M. G. and Bowyer, R. C., Approved recommendation on quality control in clinical chemistry. Part 1. General principles and terminology. *Journal of Clinical Chemistry and Clinical Biochemistry*, **18** (1980), 69–77. Part 2. Assessment of analytical methods for routine use. *Journal of Clinical Chemistry and Clinical Biochemistry*, **18** (1980), 78–88. Part 3. Calibration and control materials. *Journal of Clinical Chemistry and Clinical Biochemistry*, **18** (1980), 855–860. Part 4. Internal quality control. *Journal of Clinical Chemistry and Clinical Biochemistry*, **21** (1983), 877–884. Part 5. External quality control. *Journal of Clinical Chemistry and Clinical Biochemistry*, **21** (1983), 885–892. Part 6. Quality requirements from the point of view of health care. *Journal of Clinical Chemistry and Clinical Biochemistry*, **18** (1980), 861–866.

10. Hansert, E. and Stamm, D., Determination of assigned values in control specimens for internal accuracy control and for interlaboratory surveys. Evaluation of 200 different lots with identical experimental design. Experience and conclusions. *Journal of Clinical Chemistry and Clinical Biochemistry*, **18** (1980), 461–490.

11. Boutwell, J. H. (Ed.), *A National Understanding for the Development of Reference Materials and Methods for Clinical Chemistry. Proceedings of a Conference*. American Association for Clinical Chemistry. Washington, D.C., 1978.

12. Westgard, J. O., Groth, T. and De Verdier, C. H., Principles for developing improved quality control procedures. *Scandinavian Journal of Clinical Laboratory Investigation*, **44** (1984) **Suppl. 72**, 19–41.

13. Regulations and explanations regarding the implementation of the guidelines of the Medical Society of West Germany. *Mitteilungen der Deutschen Gesellschaft für Klinische Chemie* **3** (1974), 33–43; *Deutsches Äzteblatt*, **71** (1974), 961–965.

14. Qualitätssicherung der quantitativen Bestimmungen im Laboratorium Neue Richtlinien der Bundesärztekammer. *Deutsches Ärzteblatt*, **85** (1988), A697–A712.

15. Westgad, J. O., Barry, P. L., Hunt, M. R. and Groth, T., A multi-rule Shewhart Chart for quality control in clinical chemistry. *Clinical Chemistry*, **27** (1981), 493–501.

